# Versatile, in-line optical oxygen tension sensors for continuous monitoring during *ex vivo* kidney perfusion†

**DOI:** 10.1039/d3sd00240c

**Published:** 2024-02-27

**Authors:** Emmanuel Roussakis, Juan Pedro Cascales, Dor Yoeli, Alexis Cralley, Avery Goss, Anna Wiatrowski, Maia Carvalho, Hunter B. Moore, Ernest E. Moore, Christene A. Huang, Conor L. Evans

**Affiliations:** a Wellman Center for Photomedicine, Massachusetts General Hospital, Harvard Medical School Charlestown Massachusetts USA erousakis@mgh.harvard.edu evans.conor@mgh.harvard.edu; b Department of Surgery, University of Colorado Denver/Anschutz Medical Campus Aurora Colorado USA

## Abstract

Integration of physiological sensing modalities within tissue and organ perfusion systems is becoming a steadily expanding field of research, aimed at achieving technological breakthrough innovations that will expand the sites and clinical settings at which such systems can be used. This is becoming possible in part due to the advancement of user-friendly optical sensors in recent years, which rely both on synthetic, luminescent sensor molecules and inexpensive, low-power electronic components for device engineering. In this article we report a novel approach towards enabling automated, continuous monitoring of oxygenation during *ex vivo* organ perfusion, by combining versatile flow cell components and low-power, programmable electronic readout devices. The sensing element comprises a 3D printed, miniature flow cell with tubing connectors and an affixed oxygen-sensing thin film material containing in-house developed, brightly-emitting metalloporphyrin phosphor molecules embedded within a polymer matrix. Proof-of-concept validation of this technology is demonstrated through integration within the tubing circuit of a transportable medical device for hypothermic oxygenated machine perfusion of extracted kidneys as a model for organs to be preserved as transplants.

## Abstract 2

We report an optical oxygenation sensor technology comprising flow cell elements paired with electronic readout devices. Successful implementation in inflow and outflow oxygenation monitoring during *ex vivo* kidney perfusion is demonstrated.

## Graphical abstract

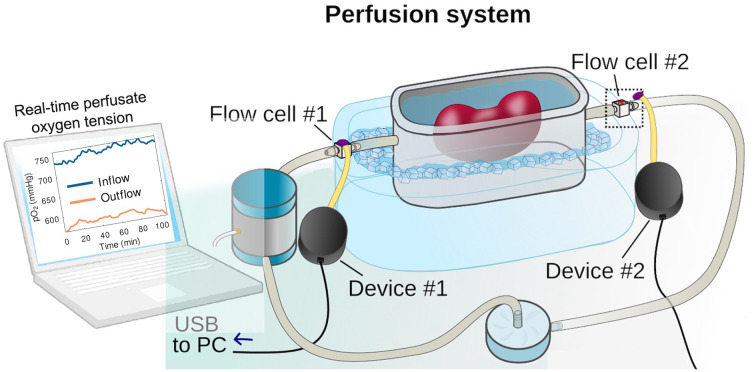

The delivery of oxygen to organs and tissues is arguably the most complex yet crucial aspect of human physiology,^1^ and oxygenation monitoring technologies are becoming increasingly essential in various branches of biomedical imaging and healthcare.^2–7^ Notably, the importance of integrating real-time sensing capabilities within technologies and systems with high translational promise and the potential to replace complex, impractical laboratory protocols has been highlighted in recent applications involving real-world physiological tissue and organ monitoring.^8–11^ An emerging field that has gained particular attention in the past decade is the preservation of organs as transplants, where technological advances in preservation technologies aim to address the continuously growing demand that necessitates the expansion of organ donor pools. Kidney donor shortage, for example, is being addressed by utilizing more marginal donor organs such as donation after cardiac death (DCD) and expanded criteria donor (ECD) kidneys.^12–14^ As these are considered higher risk organs for transplantation, it is crucial to develop methodologies for monitoring and assessing their viability during preservation which could help in predicting post-transplantation function. While there has been considerable debate on the most superior preservation strategies, there is a growing acceptance that oxygenation during perfusion is beneficial in improving kidney viability and post-transplantation outcomes.^15–18^ In addition, hypothermic machine perfusion (HMP) has been gaining broader clinical acceptance over cold static storage during the last decade.^12,19–21^ Oxygen consumption during oxygenated HMP has been shown to be among the best predictors of kidney function, showing a significant correlation with post-preservation viability.

Novel optical sensing technologies, integrated within perfusion systems and capable of automated, continuous-readout of physiological parameters have the potential to revolutionize the development and deployment of perfusion systems and transportable medical devices. Such technologies can be used in a range of applications, from sustaining life supporting organ functions in both austere environments and hospital settings following traumatic injuries to preserving organs, limbs and tissue allografts during transport for re-attachment or transplantation. Our team has been developing wearable tissue oxygenation monitoring technologies composed of novel, flexible and ultra lightweight oxygen-sensing materials and low-power, miniature electronic components. These materials rely on synthetic metalloporphyrin-based, oxygen-sensing phosphors that function on the principle of phosphorescence quenching.^22–25^

One of the challenges in designing oxygen-sensing phosphors is overcoming the problem of excessive quenching in oxygen rich environments that can dramatically reduce the materials' sensitivity. Phosphorescence quenching-based materials and techniques are typically designed to function in hypoxic and normoxic environments and they typically suffer from low dynamic range once oxygenation raises above physiological values. As a result, there have been only a handful of reports demonstrating that materials operating on the principle of phosphorescence quenching can be applied in monitoring tissue hyperoxic values.^10,11,26–29^ We have recently explored incorporation of in-house developed phosphors within various polymer films,^25,30,31^ and identified optimally performing materials capable of producing a strong, visual response to changes in oxygen partial pressures (*p*O_2_).

Here we report the development of oxygen-sensing elements in the form of miniaturized flow cells, designed for integration within perfusion tubing circuitry, and paired with prototype electronic readout devices (Fig. 1). The flow cells feature poly(propyl methacrylate) (PPMA)-based thin films with embedded metalloporphyrin phosphor molecules. In addition to bright phosphorescence that is both easily excitable and detectable by miniature, low-power electronic components,^32^ the structural features of our pivaloxy-functionalized metalloporphyrin allow for chemical compatibility with the PPMA host polymer matrix that enables encapsulation of the porphyrin with no detectable aggregation. The phosphorescence quenching principle is well known and has been successfully implemented recently in continuous tissue oxygenation monitoring in various forms, including implantable sensors.^10,11,33,34^ The optimal performance of our phosphor molecules within solid thin films expands the range of O_2_-sensing materials operating in extreme hyperoxic environments (up to 100% O_2_) without loss of accuracy, opening the way to future applications in areas such as transplant surgery. To our knowledge, applications involving multifunctional flow cells, designed as disposable elements paired with programmable readout devices that enable integration of in-line, automated and continuous perfusate oxygenation monitoring, have not yet been reported. This proof-of-concept application introduces a new methodology for implementing oxygenation monitoring within portable hypothermic machine perfusion devices built for preserving and transporting to-be-transplanted organs. This combination represents a readily translatable toolkit that can be broadly applicable to the field of physiological monitoring and can have broader implications in revolutionizing clinically used technologies and transportable medical devices with enhanced, continuous and automated monitoring capabilities. This is currently an unmet need as existing portable devices lack the sensing capabilities present in large, cart-based hospital systems equipped with sensors and monitors, and instead rely on frequent, impractical perfusate sampling for analytical testing. Notably, this work highlights the elegance of implementing synthetic chemistry and materials design in opening new avenues towards integrating powerful new optical sensing methodologies within portable systems, requiring only inexpensive, miniaturized electronic readout components rather than cumbersome and sophisticated imaging systems or monitors that have so far limited their use in such applications.

**Fig. 1:**
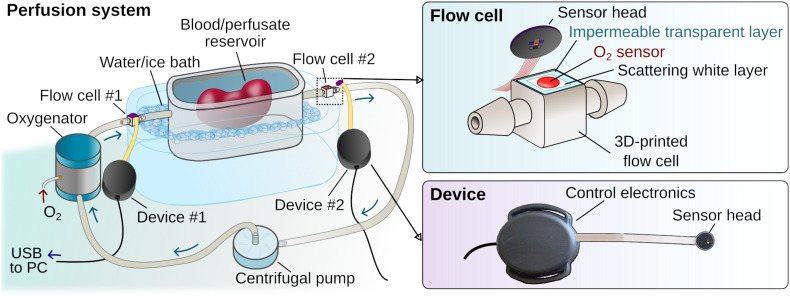
Illustration showing our prototype oxygen tension sensing device placement within an extracorporeal circulation system used for hypothermic perfusion of extracted organs. The system is composed of a temperature-controlled reservoir, a tubing circuit and an oxygenator. Oxygen tension can be measured within the flowing perfusate before and after the reservoir simultaneously. The top right drawing shows our prototype O_2_-sensing flow cell connected to the tubing of such a device, and the bottom the oxygenation readout device.

A multi-layer construction approach was used to assemble the sensing material in the form of a circular disk composed of: 1) a thin, flexible and clear polyvinyl chloride (PVC) support layer; 2) a poly(propyl methacrylate) (PPMA) polymer-based sensing film containing embedded phosphor molecules; and 3) a breathable, white silicone/titanium dioxide coating used to scatter the sensor molecules' luminescence back to the readout electronics. The rapidly responding oxygen-sensing film was formulated by embedding in-house developed phosphorescent platinum porphyrin molecules within a breathable poly(propyl methacrylate) (PPMA) host polymer matrix (see the ESI† “Materials and methods” section). A solution containing both metalloporphyrin phosphor molecules and PPMA in dichloromethane was spin-coated onto the PVC support, resulting in a thin film following solvent evaporation.

The PVC film used as a support for the sensing formulation was selected through testing flexible films of different types and thicknesses. PVC worked better than acrylic in achieving a homogeneous coating of the PPMA formulation without noticeable damage to the film by the solvent, while the 8 mil-thick PVC was chosen over thicker films in order to minimize the effect on light transmission from and to the readout device through the film (LED excitation light and generated phosphorescence, respectively). In addition, PVC has been shown to have excellent oxygen barrier properties, which is highly beneficial in blocking atmospheric oxygen from reaching the sensing layer, while it is also considered safe being among the materials approved and commonly used in manufacturing food packaging as well as the tubing lines for medical devices and applications, albeit typically coated or modified.^35–43^

To rule out any adverse effects due to the use of PVC as a support layer for the PPMA O_2_-sensing formulation, phosphorescence lifetime characterization under deoxygenated conditions was performed for a PVC-backed film as well as a stand-alone PPMA thin film. Any undesirable material mixing with PVC that would have resulted in chemical incompatibility with the phosphor molecules, would have been reflected in changes in phosphorescence lifetime. We have previously shown that the *τ*(0) is significantly decreased when the metalloporphyrin molecules are embedded within chemically incompatible materials, due to aggregation of the phosphor molecules.^31^ Here, the results showed the lifetime of the metalloporphyrin phosphor molecules within the PVC-backed sensing film (*τ*(0) = 98.2 μs) to be practically identical to that of the stand-alone PPMA film, as can be seen by the overlap of the decay curves for the two materials (ESI† Fig. S1). Remarkably, these phosphorescence lifetime values are almost identical to the inherent *τ*(0) we have reported for the metalloporphyrin molecules under ideal conditions (101 μs in a dilute, deoxygenated phosphor solution in dichloromethane).^44^

Combining the material assembly with 3D-printing technology, a prototype hollow flow cell component was created with a circular hole over which the oxygen-sensing material was affixed. The flow cell was designed with connectors that enable its integration in series within tubing circuits of perfusion systems capable of circulating physiological fluids (such as blood, perfusate, or saline buffer) from a reservoir using a centrifugal pump (Fig. 1 and ESI† Fig. S2).

To prevent contact of the coated white layer with the circulating fluid that could potentially result in the leaching of particles from the sensing material and coating into circulation, an additional layer of a medical adhesive film was placed over the white coating before affixing the material onto the flow cell. During the initial testing phase the response of our material and readout device prototype was compared against a reference *p*O_2_ monitor (PreSens Precision Sensing GmbH; Regensburg, Germany), using flow cells with and without the addition of the extra leaching-prevention adhesive layer over the multi-layered sensing material. The two devices were recording data continuously during a de-oxygenation and re-oxygenation cycle where oxygen tension in the circulating buffer was changed by flowing nitrogen gas (oxygen displacement) or oxygen, respectively. The plots of the time derivative of *p*O_2_ (Fig. 2) showed that the prototype oxygen sensor practically responds to changes in oxygenation as rapidly as the reference analytical monitor. It is worth noting that the prototype sensing material without the extra adhesive layer responded marginally faster than the reference device (Fig. 2(a) inset), while the addition of the adhesive only resulted in a negligible lag in response time compared to the reference device (Fig. 2(b) inset), calculated to be approximately 11 seconds.

**Fig. 2:**
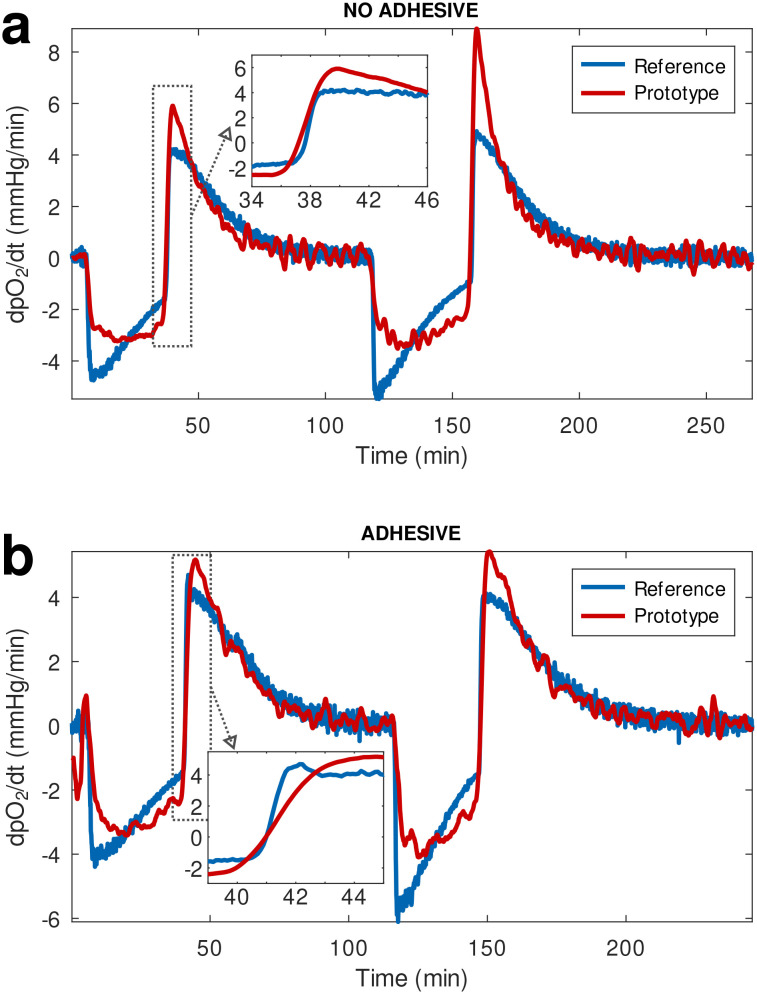
Comparison of the response time of our prototype oxygen-sensing technology to that of a commercial, reference oxygenation monitor, visualized through the time derivative of *p*O_2_. We tested oxygen-sensing materials (a) without and (b) with the addition of an extra layer of medical adhesive between our sensing layer and the perfusate. The inset in (a) shows a faster response of our device compared to the reference sensor, while the inset in (b) shows the addition of an extra layer results in a delay of 11 seconds with respect to the reference.

Using a testing and calibration system we assembled to simulate oxygenated perfusate circulation (ESI† methods and Fig. S2), we generated phosphorescence signal-*vs.-p*O_2_ calibration plots for the materials (ESI† Fig. S3). We further analyzed the data to obtain information on the performance of our sensor prototype (see ESI† section “Sensitivity and limit of detection”, and Fig. S4 and S5). Oxygenation was changed across a broad *p*O_2_ range, from physiological to hyperoxic, and temperature was set between 7 and 8 °C to mimic the conditions used in hypothermic oxygenated machine perfusion applications. Phosphorescence signal originating from the oxygen-sensing material was recorded by custom-built electronic sensor devices,^32,45^ while independent *p*O_2_ values were simultaneously being measured by a commercial analytical oxygen monitor as a reference.

The response of the oxygen sensing disk affixed onto the 3D-printed flow cell was evaluated by monitoring oxygen-dependent changes in phosphorescence as a function of time both in terms of emission intensity and lifetime. Results from both modalities show a similar, rapid response of the sensing material to changes in oxygen tension induced within the tubing of the testing system while flowing a buffer solution. As these sensors can readily operate in any type of liquid flow environment, they can additionally be applied to perfusion systems for *ex vivo* monitoring of the to-be-transplanted organs.

In a proof-of-concept application, the flow cell and electronic device prototype was validated in the continuous, 2 h-long monitoring of perfusate during *ex vivo* oxygenated hypothermic machine perfusion of pig kidneys within a transportable organ preservation system (Kidney Assist Transport; XVIVO Perfusion, Göteborg, Sweden). The short duration of the *ex vivo* kidney perfusion experiment is consistent to previously reported randomized clinical trials comparing the effects of the two methods widely used to preserve donor organ in clinical, kidney transplant settings (hypothermic oxygenated machine perfusion *vs.* cold storage), for extended criteria donor kidneys.^46,47^ Typically, the protocols reported in these studies involved a short term reconditioning of kidneys using oxygenated HMP, after an initial period of static cold storage. In fact, results from post-transplantation outcomes have indicated that prolonging the cold ischemia time (>10 h) yielded no benefit in long-term graft survival.^47–49^ It should be noted that the duration of our experiment was not due to a limitation in the stability or the performance of the material, as the PPMA-based sensing film is highly thermally- and photo-stable as we have shown previously.^31^ Specifically, PPMA sensing films remained stable during autoclaving, while in spectroscopic studies they displayed less than 20% photobleaching upon continuous illumination for a period of nearly 3 hours under conditions where the power of the incident excitation light was set approximately to that of the micro-LED within the readout device. In the device experiments presented here, the material is exposed to a micro-LED flash of less than 200 ms once every 5 seconds, sinusoidally modulated at 1.6 kHz, such that the actual LED ON time is effectively 100 ms. Under these device illumination settings, the duration of continuous perfusion experiments can easily be extended for several days and beyond if required.

As already mentioned, calibrations were performed at *p*O_2_ levels ranging from physiological (100 mmHg) to hyperoxic (600 mmHg) and at hypothermic temperatures in order to generate calibration parameters that would allow us to compensate for changes in both these variables. After applying calibration parameters to the *ex vivo* perfusion monitoring data to compensate for temperature variations experienced both by the material and the electronic readout sensors, we ascertained that the oxygen-sensing material operated well within the 3D-printed flow cells connected in series to the device tubing both entering and exiting the organ reservoir (inflow and outflow, respectively), demonstrating the prototype's capabilities in monitoring perfusate oxygenation changes within the system in real time (Fig. 3).

**Fig. 3:**
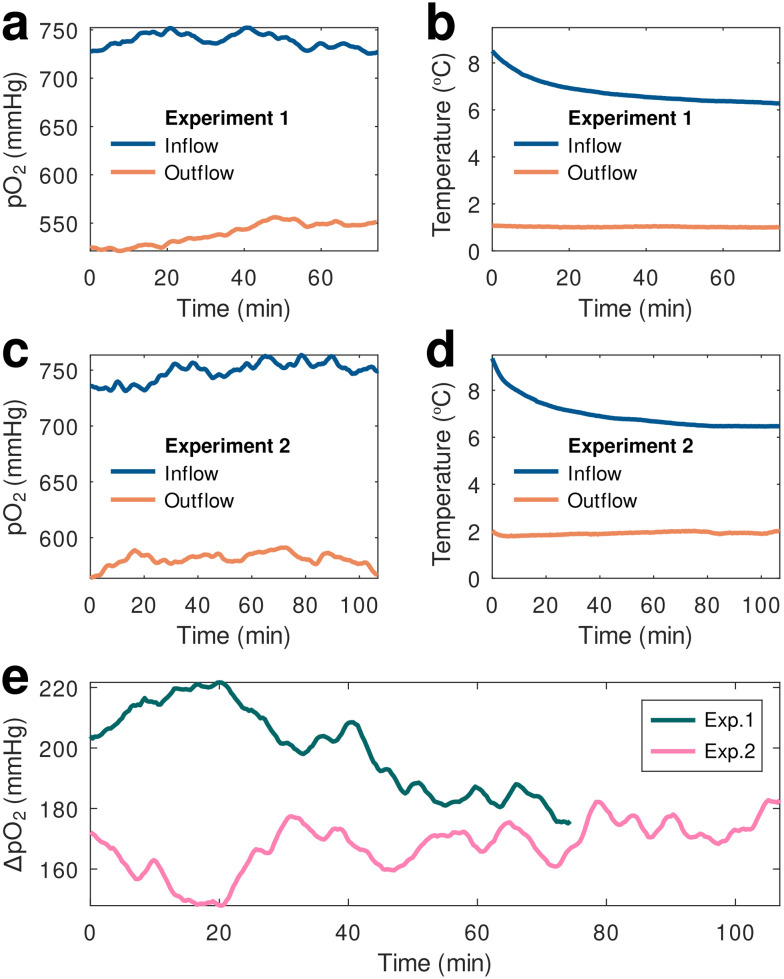
*p*O_2_ (a) and (c) and temperature (b) and (d) values from two different experiments recorded simultaneously by 2 prototype oxygen-sensing devices within a hypothermic perfusion machine containing an extracted pig kidney. The devices were placed immediately after the oxygenator (inflow data) and after the organ reservoir (outflow). (e) A *p*O_2_ drop of roughly 200 mmHg was detected in the perfusate flowing out of the reservoir with respect to the inflow value.

Two separate, continuous *ex vivo* monitoring experiments were conducted using freshly harvested kidneys immersed within the perfusate inside the hypothermic oxygenated perfusion machine's reservoir. During both experiments the *p*O_2_ of the perfusate immediately after the oxygenator (inflow) and past the organ reservoir (outflow) were simultaneously recorded using two of the prototype oxygen sensing devices. The temperature-compensated results demonstrated consistency between the two measurements showing a distinct oxygen depletion in the outflow perfusate coming from of the organ reservoir. Notably, the measurement of the oxygenated perfusate (inflow) was in complete agreement with the Kidney Assist Transport device specifications, highlighting an integrated, oxygen transfer system designed to optimally provide continuous, full and active perfusate oxygenation *via* a small medical-grade oxygen cylinder enclosed within the system. These measurements showed a steady inflow oxygenation of practically 100% (the theoretical *p*O_2_ value at 100% oxygenation under a pressure of 1 atm is 760 mmHg), during a 2 h measurement period.

In conclusion, the development of novel optical sensing modalities for blood gases and physiological analytes shows considerable promise in meeting the demands of both research and clinical applications. Continuous, automated oxygenation monitoring technologies could be widely applicable in areas of developing synthetic tissue models, preserving tissue allografts, and donated organs alike. In the field of surgical transplantation, in particular, such technologies are critically needed especially given the continuously widening gap between the availability of donated organs and the demand for transplants. However, a major challenge in incorporating real-time sensing elements within portable perfusion systems and to-be-transplanted organ preservation devices is avoiding additional components that will add to both the weight and complexity of the systems, or will require tedious data analysis and interpretation by the operator. The oxygen-sensing technology presented here can be readily integrated into a lightweight, portable toolkit for integration within transportable devices for hypothermic oxygenated machine perfusion. The ability to continuously report inflow and outflow perfusate oxygenation simultaneously has the potential to provide real-time heads-up monitoring of organ perfusion and viability, and can be readily extended for both closed-loop feedback control and automated data processing and reporting. The simplicity and minimal cost of these devices also opens the door for perfusion systems that can go beyond the hospital and first-world medical center setting towards third-world and austere environments to provide life-supporting functions under a wide array of clinical scenarios.

## Author contributions

E. R.: conceptualization, data curation, formal analysis, investigation, methodology, project administration, supervision, writing – original draft; J. P. C.: conceptualization, data curation, formal analysis, investigation, methodology, software, writing – original draft; D. Y.: investigation, methodology, writing – review & editing; A. C.: investigation, methodology, resources; A. G.: investigation, methodology; A. W.: investigation, methodology, writing – review & editing; M. C.: investigation, methodology, writing – review & editing; H. B. M.: investigation, methodology; E. E. M.: methodology, resources, supervision; C. A. H.: methodology, resources, supervision; C. L. E. conceptualization, funding acquisition, methodology, project administration, supervision, writing – review & editing.

## Conflicts of interest

E. R. and C. L. E. are inventors on patents US10905780B2 and US11253613B2.

## Supplementary Material

SD-003-D3SD00240C-s001

SD-003-D3SD00240C-s002
