# Supplementary material for: Versatile, in-line optical oxygen tension sensors for continuous monitoring during ex vivo kidney perfusion
Source: Sens Diagn. 2024 Feb 27;3(6):1014–9. doi: 10.1039/d3sd00240c (PMC11170683; doi:10.1039/d3sd00240c)
Supplement: SD-003-D3SD00240C-s001 [file SD-003-D3SD00240C-s001.pdf]

Electronic Supplementary Material (ESI) for Sensors & Diagnostics.
This journal is © The Royal Society of Chemistry 2024

Supporting Information

Versatile, in-line optical oxygen tension sensors for continuous monitoring during ex vivo kidney perfusion

Emmanuel Roussakis,[*,†] Juan Pedro Cascales,[†] Dor Yoeli,[‡] Alexis Cralley,[‡] Avery Goss,[†] Anna Wiatrowski,[†] Maia Carvalho,[†] Hunter B. Moore,[‡] Ernest E. Moore,[‡] Christene A. Huang,[‡] and Conor L. Evans[*,†]

†*Wellman Center for Photomedicine, Massachusetts General Hospital, Harvard Medical School, Charlestown, Massachusetts, United States of America*

‡*Department of Surgery, University of Colorado Denver / Anschutz Medical Campus, Aurora, Colorado, United States of America*

E-mail: erousakis@mgh.harvard.edu; evans.conor@mgh.harvard.edu

Materials and Methods

Oxygen-sensing material and flow cell

The pivaloxy-functionalized Pt-porphyrin embedded within a thin, polymer-based film as the oxygen-sensing phosphor was synthesized according to a previously published protocol.[1] The phosphor molecules were embedded within a thin, polymer-based film and coated with a white silicone/titanium dioxide scattering layer as has been previously published.[2,3] From a solution containing 90μM of the porphyrin and 50mg/mL of poly(propyl methacrylate) (PPMA) in dichloromethane, 10μL were cast on an 8mil-thick (0.008") flexible and clear polyvinyl chloride (PVC) film via spin coating. The spin coater (WS-650-23 model; Laurell Technologies) parameters were set to 500rpm with an acceleration of 5rpm/s, for 2min.

The fast-curing titanium dioxide pigment/dimethylsiloxane copolymer mixture (containing curing retarding agent and a Pt catalyst),[3] was prepared and quickly applied over the porphyrin/PPMA sensing layer by spreading into a thin film with the help of a small glass rod. Curing was completed inside an oven set to 60°C, for approximately 5min. Finally, a breathable layer of medical adhesive (3M; Medical Transfer Adhesive 1524) was applied over the white silicone coating to prevent contact with the circulating perfusate. A ring of double-sided tape was used to firmly attach the multi-layer oxygen-sensing film over a circular hole on a 3D-printed flow cell.

To rule out potential adverse effects on the performance of the metalloporphyrin phosphor molecules embedded within the PVC-backed, multi-layer film, phosphorescence lifetime characterization was performed under deoxygenated conditions (see Figure S1), and the results were compared to those of a stand-alone thin PPMA film prepared as has been previously reported.[2] The samples were cut into circular pucks using a 3mm hole punch and placed diagonally inside a septum-capped quartz cuvette using a 3D-printed spoon-like holder (Figure S1, Photo inset), in order for the sample to be aligned with the spectrometer's excitation beam. Deoxygenation was achieved by running compressed nitrogen gas through the cuvette

using a needle inlet and outlet. Phosphorescence decays were acquired using an FLS1000 steady state and phosphorescence lifetime spectrometer (Edinburgh Instruments; Livingston, UK).

The miniaturized flow cell design was based off of a straight tubing connector (McMaster-Carr; part number 5117K46), with an inner diameter of 1/4". The straight tubing connector was widened at the center to a 0.4" x 0.35" area to allow the addition of a hole with a diameter of 0.32" with a depth of 0.29". This modification allows a secure seal of the oxygen sensing film to the flow cell and direct exposure to the perfusate. A second version of the flow cell was designed with an internal diameter of 3/8". Both versions of the flow cell were printed on a Formlabs 3B printer in a white photo-polymer resin and washed in a 100% isopropyl alcohol sonic bath for 10 minutes. The flow cell was then cured in a Formcure UV oven at 60°C for 30 minutes. Supports and burrs were removed from surface of part. Special attention was paid when cleaning the internal cavity of the flow cell to ensure it was clean of uncured resin.

Electronic readout devices

The readout of phosphorescence lifetime and intensity was carried out with a wearable device previously developed in our group to continuously measure transcutaneous oxygenation.[4] As seen in Figure 1 in the main article, the device consists of a small sensor head and the main control electronics.

The control electronics consist of custom PCBs hosting a fast *analog-to-digital coverter* (ADC) chip, transimpedance amplifier and signal conditioning block, built around a wireless-capable microcontroller board (Particle Photon).

The small sensor head (14 mm diameter, 3 mm thick) is affixed on top of the flow cell to probe the oxygen-sensing material. It is composed of a 3D-printed case, a flexible printed circuit board (PCB) and small surface-mount electronics: two high power, 385 nm excitation UVA LEDs (Lumileds), a PIN photodiode (Osram) a thermistor (TDK, Tokyo, Japan) to

measure temperature.

The LED emission is modulated by a sine-wave at a frequency of 1.6kHz. The emission is filtered by a 400 nm short-pass filter composed of two ultra-thin flexible optical notch-filters. At the photodiode interface, the LED signal is blocked by placing a 500 nm long-pass filter over the PIN photodiode, by combining a flexible 405 nm long-pass filter and a polyamide film (Kapton tape, 3M).

Calibration system setup

Calibrations were performed using a mock testing system we assembled through adaptation of a previously published protocol,[5] to simulate oxygenated perfusate circulation. Our system (see Figure S2) consists of a 2L sealed water-jacketed glass reservoir where the lid was equipped with inlet/outlet ports for buffer circulation and gas bubbling, a centrifugal pump drive and pump head (Harvard Apparatus, Holliston MA, USA), connected via a tubing circuit. Our prototype oxygen-sensing flow cell element was connected in series to the tubing, along the flow of the buffer. The analytical pO_2 device (PreSens Precision Sensing GmbH; Regensburg, Germany) was placed next to our prototype, using a second flow cell where the commercial oxygen sensor spot was attached onto a square piece of PVC affixed over the circular hole of the flow cell, with the signal being recorded by the PreSens monitor in optical fiber mode. Cycles increasing and decreasing oxygenation within the circulating phosphate buffered saline (PBS) were achieved by bubbling compressed oxygen or nitrogen gas, respectively, through the reservoir lid ports. The pO_2 range during calibration was changed from physiological to hyperoxic values (approximately 100 to 600 mmHg) (Figure S3). To mimic the conditions of the *ex vivo* experiment during calibrations, the temperature of the buffer was set to 7-8°C by running cooled water through the double-wall of the jacketed reservoir using the water chiller. Additionally, ice-filled plastic bags were used during calibration in order to compensate for the electronic sensor-heads of the readout devices experiencing different temperatures due to the presence of a metal ice container over one of the sensors

within the transportable kidney perfusion device. Specifically, within a single calibration session, the cycle of increasing buffer pO_2 was performed with the electronic readout sensors allowed to experience buffer temperature, while ice-filled plastic bags were placed over the sensors during the reverse cycle (decreasing pO_2). The code running the devices and the PreSens logging software were run simultaneously, automatically recording data every 5s over a period of 7 hs (Figure S3), creating the raw data files used in generating the calibration parameters for the kidney perfusion experiments.

Ex vivo kidney perfusion experiment setup

The kidneys used for the *ex vivo* perfusion experiment were harvested from a combat casualty relevant dismounted complex blast injury (DCBI) swine model, following euthanasia.[6] Specifically, the kidneys were procured via ultra-rapid laparotomy and cannulation of the infra-renal aorta and cross-clamping of the supra-celiac aorta. The inferior vena cava was transected at a level below the renal veins to allow for effluent drainage. The abdominal organs were flushed with at least 1 liter of Custodiol® HTK Solution (Essential Pharmaceuticals LLC) and until the effluent was clear. The abdomen was packed with ice during this process. The bilateral kidneys were then removed from the body, placed in a container filled with HTK solution, which was then kept on ice until initiation of perfusion. HTK is a commercially available organ preservation solution designed to minimize ischemic injury to solid organ allografts during static cold storage. Since all interventions were done after animal euthanasia, these procedures were not subject to IACUC review but were approved by the University of Colorado Office of Laboratory Animal Resources. The kidneys were perfused with Xvivo© Kidney Assist Transport device, which perfuses the organs with oxygenated, hypothermic Belzer MPS® UW machine perfusion solution. MPS® is a commercially available organ preservation solution used clinically for kidney hypothermic perfusion preservation. Once the circuit was primed, the renal artery was connected to the arterial cannula and inserted into the cartridge, which was then connected to the machine. The

perfusion was pressure regulated at a preset perfusion pressure of 24 mmHg. The perfusion solution was oxygenated with 100% oxygen delivered at a rate of 0.1 L/min. The solution was maintained at 8°C. The organs were perfused continuously for 2 hours.

Data processing

The change in the phosphor's lifetime τ and intensity I depend on oxygen following the Stern-Volmer relation:

$$\frac{X_0}{X} = 1 + K_{SV} \cdot pO_2 + X_{off} \tag{1}$$

with $X = (\tau, I)$, X_0 the lifetime/intensity in the absence of oxygen, K_{SV} the Stern-Volmer quenching constant and X_{off} and offset due to the phosphorescence of porphyrin molecules shielded from oxygen.[7] To compensate for changes in temperature, we employed a K_{SV} coefficient that is linearly-dependent on temperature[3,4] ($K_{SV} = K_0 + K_1 \cdot T$), which accounts for the trends observed in the data (see Supplementary Figure S3).

An algorithm based on Multiple Linear Regression in the matrix form[8] was developed using GNU Octave[9] to extract lifetime and intensity of the emission.[4]

As is stated in the calibration section, an analytical pO_2 device (PreSens) is used as reference sensor to monitor the pO_2 of the fluid during calibration measurements. These reference pO_2 values are used to fit the dependence of lifetime or intensity with oxygenation, which follow the Stern-Volmer relation above with a GNU Octave script and a non-linear regression algorithm.[4] We are then able to extract calibration parameters X_0, K_0 and K_1 calibration parameters for each device.

PVC-backed oxygen-sensing film characterization

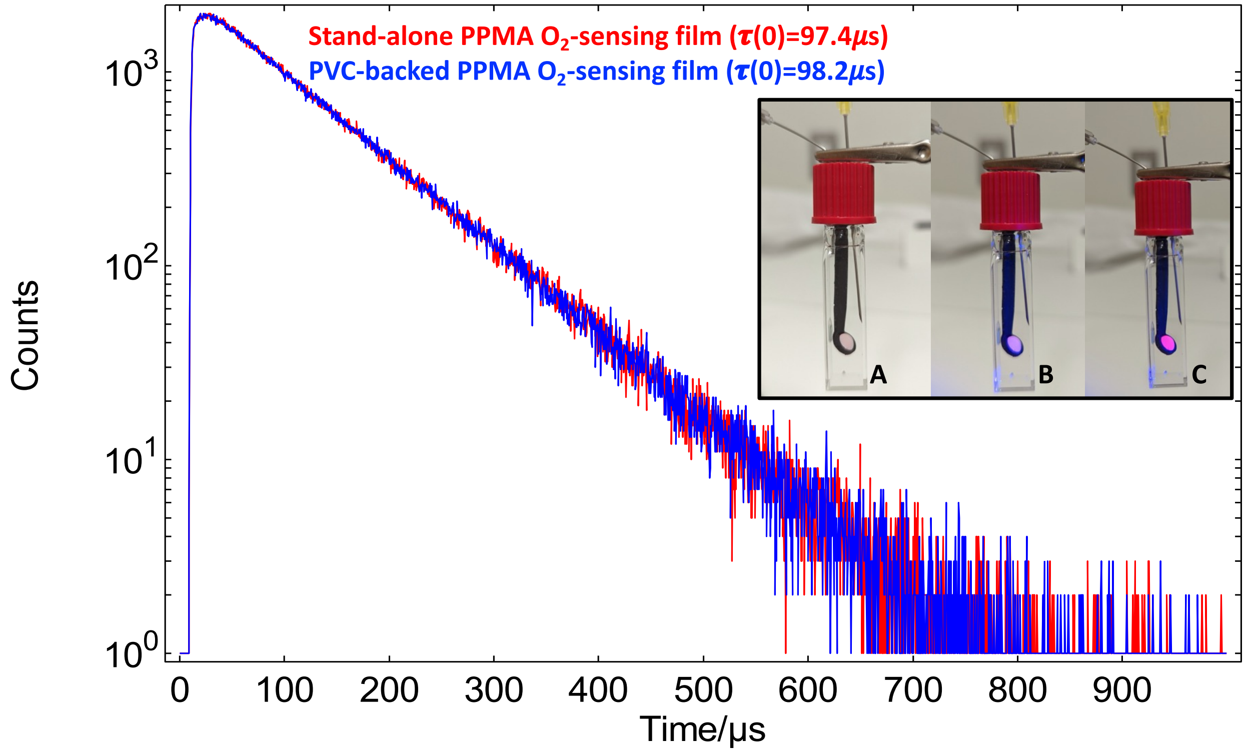

Figure S1: Comparison of the metalloporphyrin phosphor molecules' lifetime decay curves within a PVC-backed film versus a stand-alone PPMA film, under deoxygenated conditions ($\tau(0)$). Photo inset: setup showing the placement of a 3mm circular, PVC-backed multi-layer sensing film (PVC support layer facing up) inside a septum-capped quartz cuvette using a 3D-printed spoon-like holder. (A) photo taken under ambient air and light conditions (no excitation); (B) photo under ambient air conditions, while the film was illuminated with a handheld, blue LED flashlight; (C) photo under deoxygenated conditions (compressed nitrogen gas purge), while the film was illuminated with a handheld, blue LED flashlight.

Calibration system setup

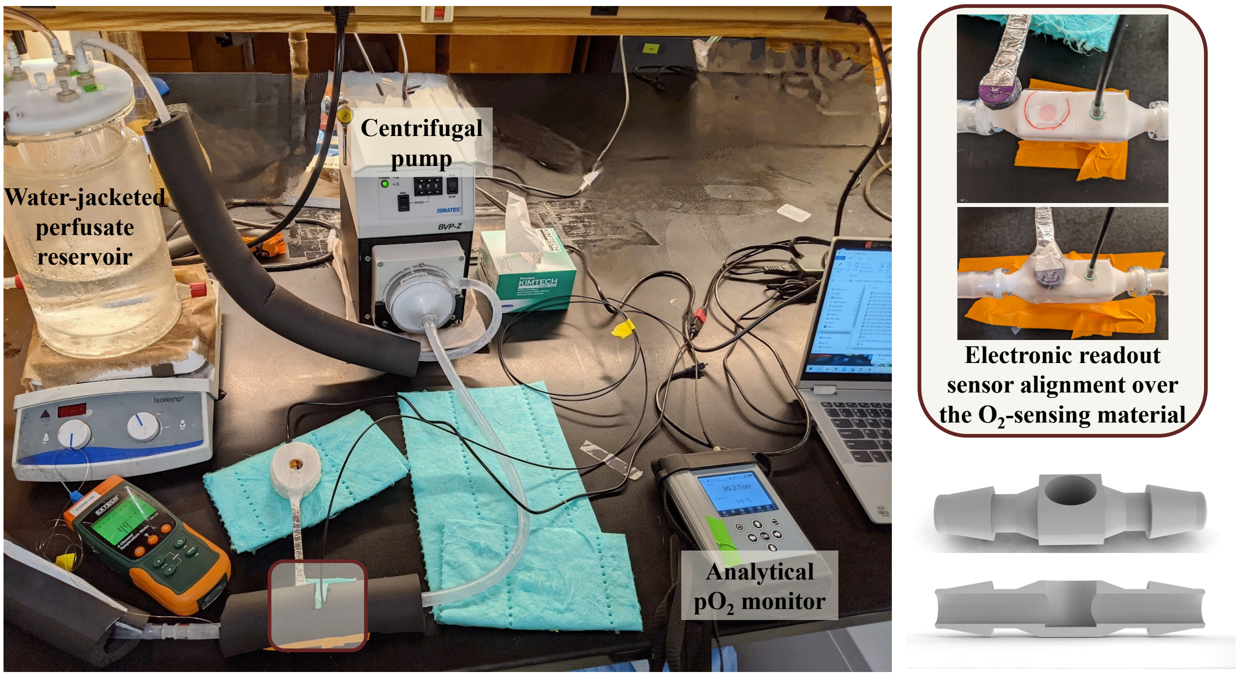

Figure S2: Left: A photo showing the testing and calibration system, consisting of a 4L water-jacketed reservoir connected to a water chiller for temperature control, a centrifugal pump and tubing circuitry. It was assembled to circulate buffer under conditions that simulate oxygenated machine perfusion systems. The PVC-backed, PPMA-based oxygen sensing material as well as the analytical reference sensor dot were affixed within flow cells connected in series to the tubing, with the electronic sensor-head centered over the circular oxygen-sensing material (zoomed-in panel; top right). Bottom right: full and sectional view of the miniaturized, 3D-printed flow cell version, designed to fit within the tubing slot of the Kidney Assist Transport without extending the overall tubing circuitry length that would affect the device function.

Calibration data

As was described previously,[4] we can obtain two estimates of pO_2, derived from the lifetime and the intensity of the phosphorescence emission of our oxygen-sensing film.

The dependence of the lifetime τ and intensity I with oxygen is governed by the Stern-Volmer relation,[10] which we have modified to account for changes with temperature.[4] For a measured variable C, which could be τ or I, we have:

$$C = C_0/(1 + K_{eff} \cdot pO_2) + C_{OFF}$$

where C_0 is the value of C in the absence of oxygen and C_{OFF} is a non-oxygen dependent offset. The temperature dependence is built into the equation as the Stern-Volmer quenching constant, K_{eff}, is temperature dependent due to changes in the oxygen-diffusion parameters of the film. A linear dependence of K_{eff} with temperature ($K_{eff} = K_0 + K_1 \cdot T$) accounts for the trends observed.[11]

Therefore, fitting the mentioned Stern-Volmer relation to lifetime or intensity data, we obtain 4 calibration parameters w_i with $i \in [1, 4]$ corresponding to C_0, K_0, K_1 and C_{OFF}.

The following figure (Fig. S3) presents calibration data carried out prior to the *ex vivo* studies. As seen in the X axis of the plots of Fig. S3 (b) and (e), oxygenation was changed across a broad pO_2 range, from physiological to hyperoxic, and temperature was set between 7 and 8°C to mimic the conditions used in hypothermic oxygenated machine perfusion applications. Fig.S3(a),(b) and (d),(e) show that features in lifetime and intensity data due to changes in pO_2 and temperature, respectively, are well described by the temperature dependent Stern-Volmer equation.

The estimate of pO_2 obtained from lifetime and intensity data, shown in Fig.S3(c) and (f), closely matches what is measured with a reference commercial pO_2 sensor, with the small discrepancies thought to arise from a difference in response time of both devices and from each sensor experiencing different temperature gradients due to their location within

the calibration system.

Lastly, we obtained incorrect readings using our sensor roughly between 180 and 215 minutes (shadowed region in Fig.S3 (c) and (f)) , which were due to the accumulation of air bubbles in our flow cell right below the area where the O_2 sensing film is located. The accumulation of these bubbles was noted during the experiment and the data points between 180 and 215 minutes were not used for the fitting of the Stern-Volmer equation and thus do not influence the calibration parameters. As can be seen from the extensive temperature and oxygen range used, the exclusion of these small set of data points did not influence the overall calibration of the device. Future work will involve including a bubble trap in our system to avoid this kind of issue in the future.

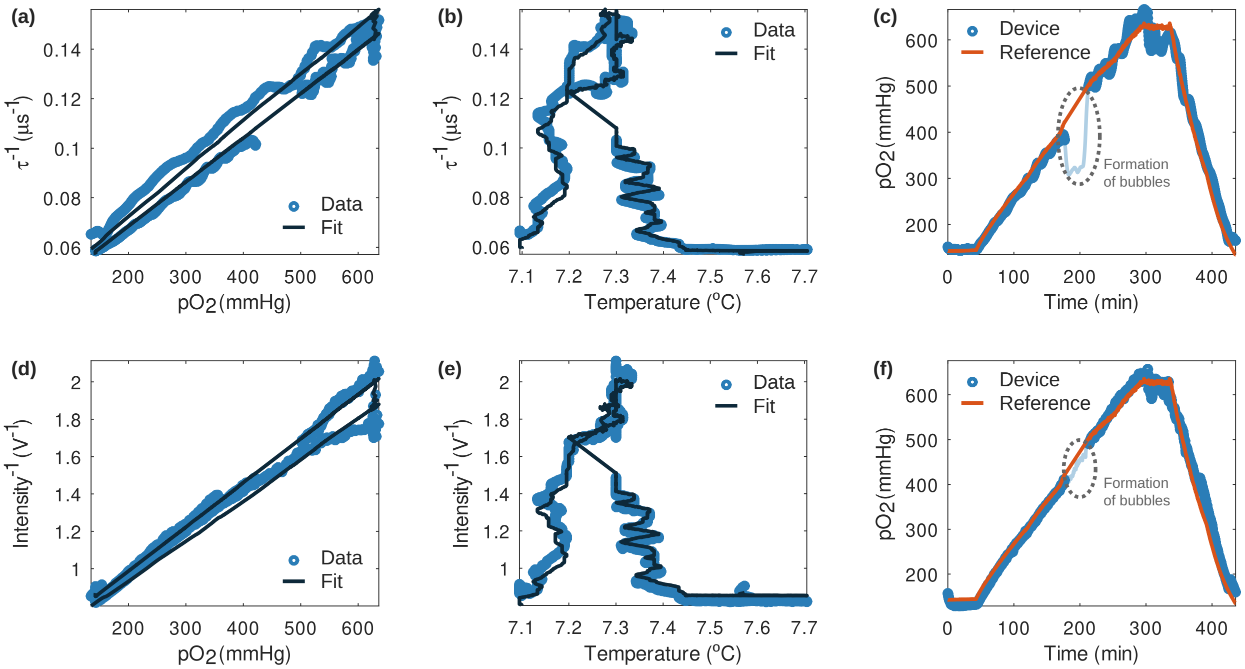

Figure S3: Response of the oxygen sensing film in a flow cell to pO_2 and temperature during a calibration run along with a fit of the temperature dependent Stern-Volmer equation. (a) Stern-Volmer plot of lifetime data and its fit to the temperature dependent Stern-Volmer equation. The model is able to describe the variation of the measured lifetime with changes in partial pressure of oxygen and temperature. (b) Complementary lifetime data vs temperature graph. (c) Estimated pO_2 measured by our device and a commercial reference sensor. (d), (e) and (f) show equivalent plots for the intensity data revealing similar features.

Sensitivity and limit of detection

To calculate the error in our pO_2 estimates, we have taken the following steps:

- The standard error of the calibration coefficients is obtained from the variance-covariance matrix of the fitting parameters w_i, which we label σ_{w_i}.
- The lifetime is obtained by measuring a phase difference θ between the excitation signal and the measured phosphorescence, via the equation $tan\theta = 2\pi\tau f$ with f the modulation frequency of the excitation LEDs. Each time the pO_2 is sampled, we obtain the standard error of θ and the intensity I (σ_τ and σ_I) from the variance-covariance matrix of a multiple-linear regression fit.[4] The error in lifetime is obtained through error propagation:

$$\sigma_\tau = |\frac{sec^2\theta}{2\pi f}|\sigma_\theta$$

- The standard error of the lifetime or intensity derived pO_2 was calculated by using the Stern-Volmer equation and error propagation, parting from:

$$pO_2 = \frac{1}{K_{eff}}\left(\frac{w_1}{C - w_4} - 1\right)$$

Let τ or I be C, then:

$$\sigma^2_{pO_2^C} = \sum_i |\frac{\partial pO_2^C}{\partial x_i}|^2 \sigma^2_{x_i}$$

with the x_i being C (lifetime or intensity), temperature and the four fitting coefficients w_i obtained from the calibration. The error in temperature was obtained by calculating the standard deviation of a 50 point temperature reading during which temperature was not changed ($\sigma_T = 0.004$ °C). The different terms are:

$$\frac{\partial pO_2^C}{\partial C} = \frac{1}{K_{eff}} \frac{w_1}{(C - w_4)^2}$$

$$\frac{\partial pO_2^C}{\partial T} = \frac{w_3}{K_{eff}^2} \left[\frac{w_1}{(C - w_4)} - 1 \right]$$

$$\frac{\partial pO_2^C}{\partial w_1} = \frac{1}{K_{eff}} \frac{1}{(C - w_4)}$$

$$\frac{\partial pO_2^C}{\partial w_2} = \frac{1}{K_{eff}^2} \left[\frac{w_1}{(C - w_4)} - 1 \right]$$

$$\frac{\partial pO_2^C}{\partial w_3} = \frac{T}{K_{eff}^2} \left[\frac{w_1}{(C - w_4)} - 1 \right]$$

$$\frac{\partial pO_2^C}{\partial w_4} = -\frac{1}{K_{eff}} \frac{w_1}{(C - w_4)^2}$$

Since the signal amplitude changes with pO_2, we obtain an experimental error which improves as oxygen partial pressure decreases, as can be seen in Fig. S4

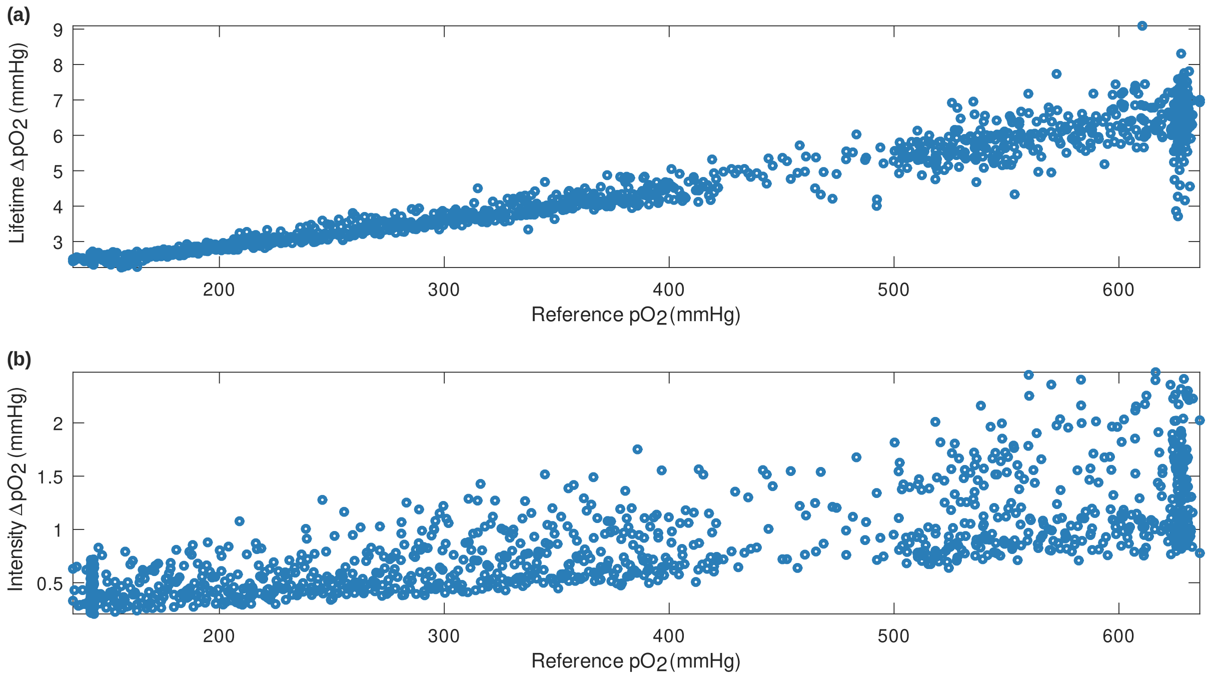

Figure S4: Experimental error in (a) lifetime and (b) intensity estimates of pO_2 as a function of the oxygen partial pressured measured with a reference sensor.

Sensitivity

Because τ and I depend non-linearly on pO_2, the sensitivity depends on the oxygen partial pressure. We define the dynamic sensitivity for quantity C as $|\frac{dC}{dpO_2}|$ (with C being τ or I). Plotting the sensitivity for lifetime and intensity, as seen in Fig. S5, we obtain the expected dependence, sensitivity increases with decreasing oxygenation.

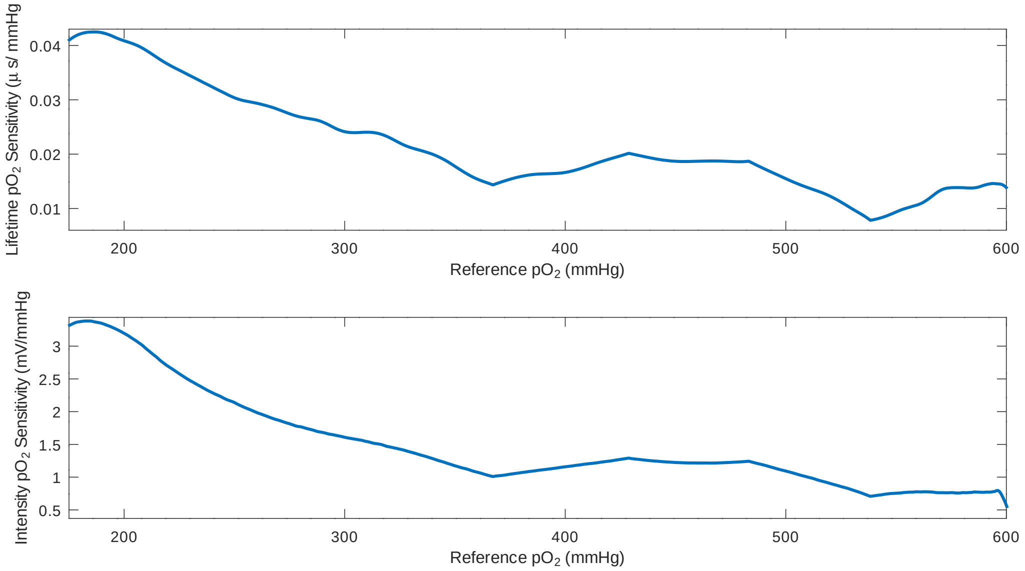

Figure S5: Dynamic sensitivity of the lifetime and intensity based pO_2 detection.

Limit of detection

If we define the limit of detection (LOD) of our devices as the 95% confidence interval, defined as $95\%CI = 2\sigma_{pO_2}$ with σ_{pO_2} the obtained error shown in Fig. S4. The trend of both graphs is approximately linear, so can then fit a linear dependence with pO_2 to the 95%CI, and we obtain:

- **Lifetime pO_2:** $LOD_\tau(pO_2) = 2.368 + 0.017 \cdot pO_2$ mmHg, which translates to a LOD of 2.37 mmHg in the absence of oxygen and 15 mmHg in an atmosphere of pure oxygen.
- **Intensity pO_2:** $LOD_I(pO_2) = 0.195 + 0.004 \cdot pO_2$ mmHg, which translates to a LOD of 0.2 mmHg in the absence of oxygen and 3 mmHg in an atmosphere of pure oxygen.

With regards to the real-world application of the device, our limit of detection depends on the range of oxygen partial pressure being measured, which in the best/worst case scenario is of 2-15 mmHg for lifetime and 0.2-3 mmHg for intensity. The intensity derived metric, although more sensitive, is prone to measurement errors due to relative motion between detector and O_2 sensing film, photobleaching, etc. The results obtained are in line, for example, with the commercial Periflux 6000 used for transcutaneous oxygen sensing (gas only, and not in fluid), which has an accuracy of 5 mmHg only in the range 5-200 mmHg, while our device extends up to 1 atmosphere of oxygen.

References

(1) Roussakis, E.; Li, Z.; Nowell, N. H.; Nichols, A. J.; Evans, C. L. Bright, "Clickable" Porphyrins for the Visualization of Oxygenation under Ambient Light. *Angew. Chem. Int. Ed.* **2015**, *54*, 14728–14731.

(2) Li, X.; Roussakis, E.; Cascales, J. P.; Marks, H. L.; Witthauer, L.; Evers, M.; Manstein, D.; Evans, C. L. Optimization of bright, highly flexible, and humidity insensitive porphyrin-based oxygen-sensing materials. *J. Mater. Chem. C* **2021**, *9*, 7555–7567.

(3) Witthauer, L.; Cascales, J. P.; Roussakis, E.; Li, X.; Goss, A.; Chen, Y.; Evans, C. L. Portable Oxygen-Sensing Device for the Improved Assessment of Compartment Syndrome and other Hypoxia-Related Conditions. *ACS Sens.* **2021**, *6*, 43–53.

(4) Cascales, J. P.; Roussakis, E.; Witthauer, L.; Goss, A.; Li, X.; Chen, Y.; Marks, H. L.; Evans, C. L. Wearable device for remote monitoring of transcutaneous tissue oxygenation. *Biomed. Opt. Express* **2020**, *11*, 6989–7002.

(5) Ferrari, L.; Rovati, L.; Fabbri, P.; Pilati, F. Continuous haematic pH monitoring in extracorporeal circulation using a disposable florescence sensing element. *J. Biomed. Opt.* **2013**, *18*, 027002.

(6) Cralley, A. L.; Moore, E. E.; Kissau, D.; Coleman, J. R.; Vigneshwar, N.; DeBot, M.; Schaid, J., Terry R.; Moore, H. B.; Cohen, M. J.; Hansen, K.; Silliman, C. C.; Sauaia, A.; Fox, C. J. A combat casualty relevant dismounted complex blast injury model in swine. *J. Trauma Acute Care Surg.* **2022**, *93*, S110–S118.

(7) Lakowicz, J. R. *Principles of Fluorescence Spectroscopy*; Springer, 2006; Vol. Third Edition.

(8) Pedersen, M. S. et al. The Matrix Cookbook. *Matrix* **2008**,

(9) John W. Eaton, S. H., David Bateman; Wehbring, R. *GNU Octave version 3.8.1 manual: a high-level interactive language for numerical computations*; CreateSpace Independent Publishing Platform, 2014; ISBN 1441413006.

(10) Stern, O.; Volmer, M. Über die abklingzeit der fluoreszenz. *Phys. Z* **1919**, *20*, 183–188.

(11) Friedl, D. P. F. Investigating the Transfer of Oxygen at the Wavy Air-Water Interface under Wind-Induced Turbulence. *Thesis* **2013**,
