# Supplementary material for: Versatile, in-line optical oxygen tension sensors for continuous monitoring during ex vivo kidney perfusion
Source: Sens Diagn. 2024 Feb 27;3(6):1014–9. doi: 10.1039/d3sd00240c (PMC11170683; doi:10.1039/d3sd00240c)
Supplement: SD-003-D3SD00240C-s002 [file SD-003-D3SD00240C-s002.zip › FigS1.pdf]

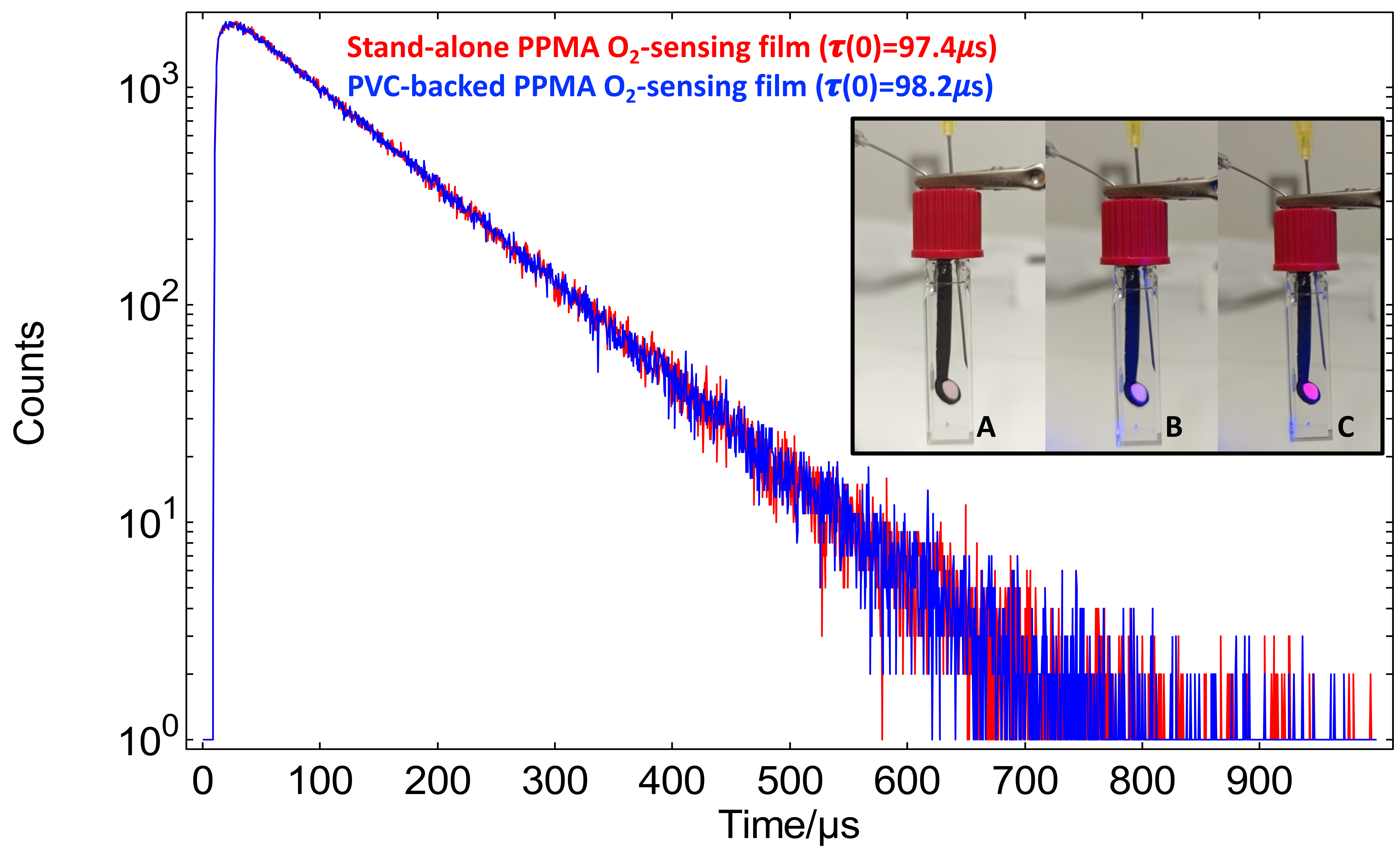

Stand-alone PPMA O_2-sensing film ($\tau(0)$=97.4μs)
PVC-backed PPMA O_2-sensing film ($\tau(0)$=98.2μs)
Counts
10^3
10^2
10^1
10^0
0
100
200
300
400
500
600
700
800
900
Time/μs
A
B
C
