# Supplementary material for: Versatile, in-line optical oxygen tension sensors for continuous monitoring during ex vivo kidney perfusion
Source: Sens Diagn. 2024 Feb 27;3(6):1014–9. doi: 10.1039/d3sd00240c (PMC11170683; doi:10.1039/d3sd00240c)
Supplement: SD-003-D3SD00240C-s002 [file SD-003-D3SD00240C-s002.zip › FigS2.pdf]

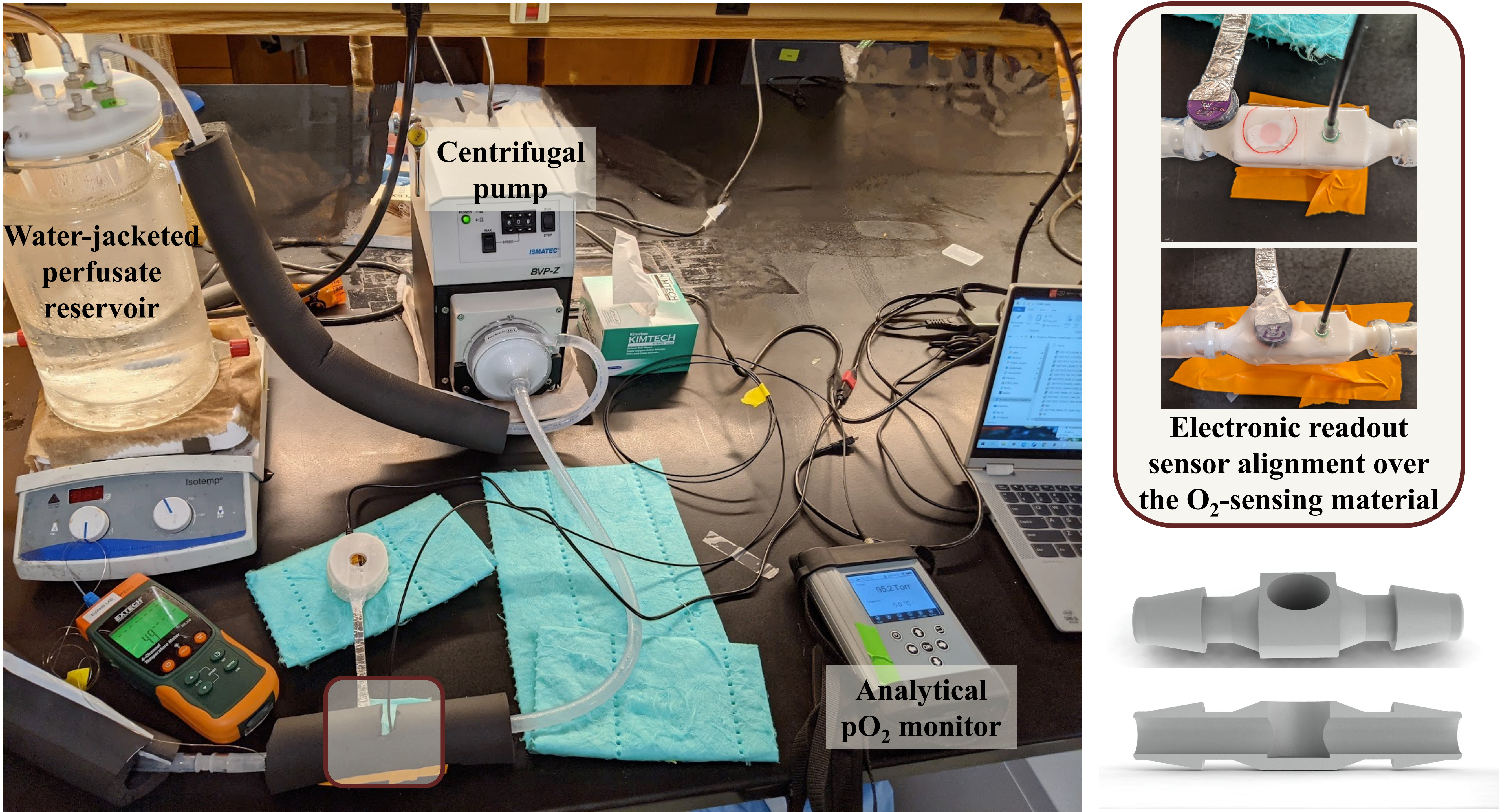
Centrifugal pump
Water-jacketed perfusate reservoir
ISMATEC
BVP-Z
KIMTECH
Isotemp
EXTECH
Electronic readout sensor alignment over the O_2-sensing material
Analytical pO_2 monitor
