# Supplementary figures and images for: Versatile, in-line optical oxygen tension sensors for continuous monitoring during ex vivo kidney perfusion
Source: Sens Diagn. 2024 Feb 27;3(6):1014–9. doi: 10.1039/d3sd00240c (PMC11170683; doi:10.1039/d3sd00240c)

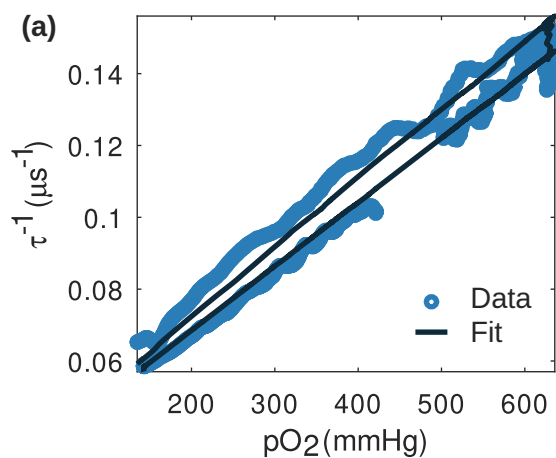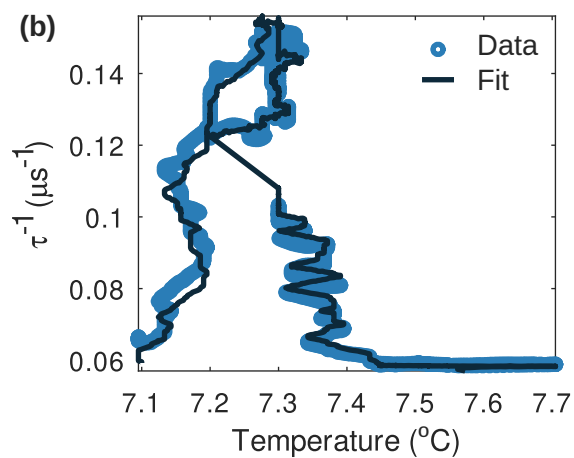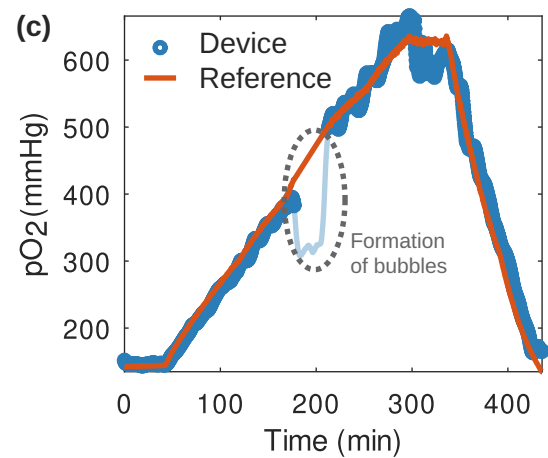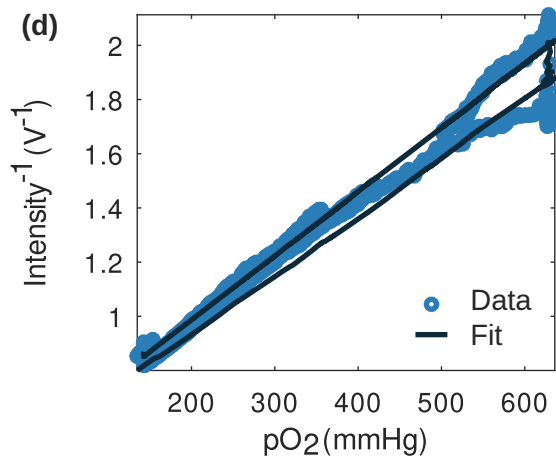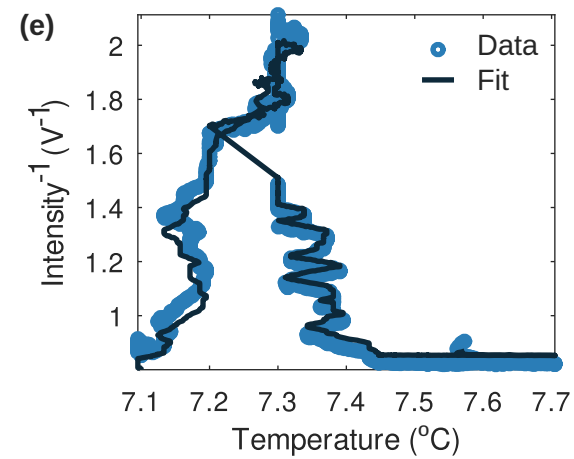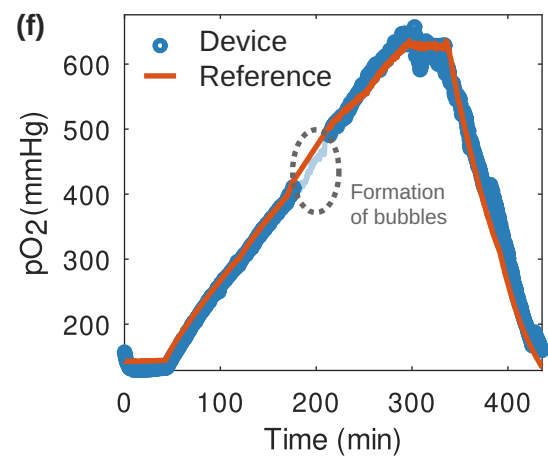

Supplement: SD-003-D3SD00240C-s002 [file SD-003-D3SD00240C-s002.zip › FigS3.pdf]

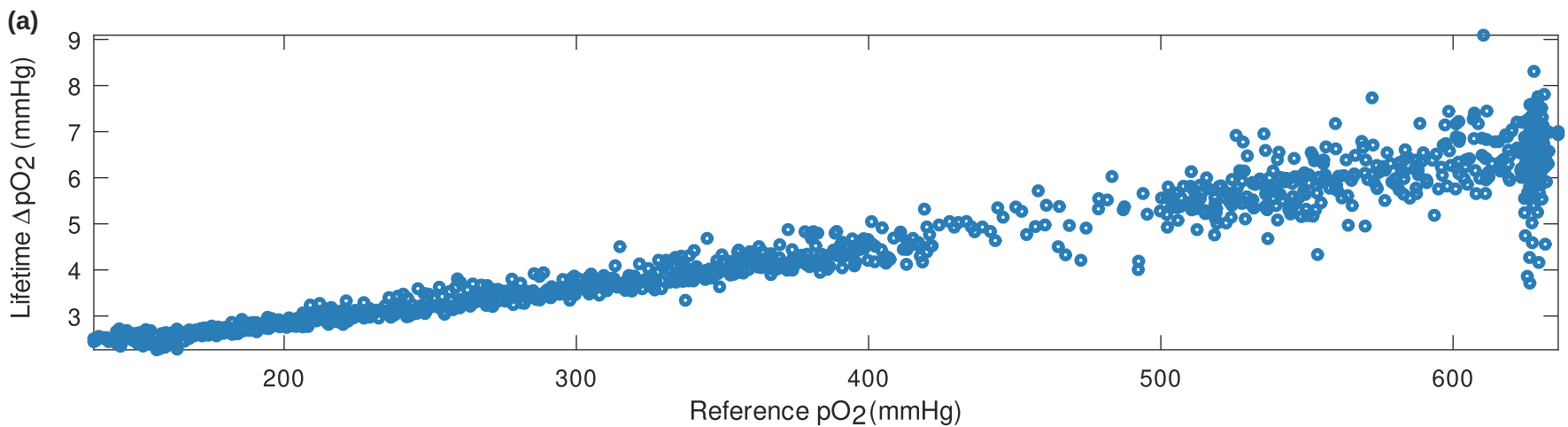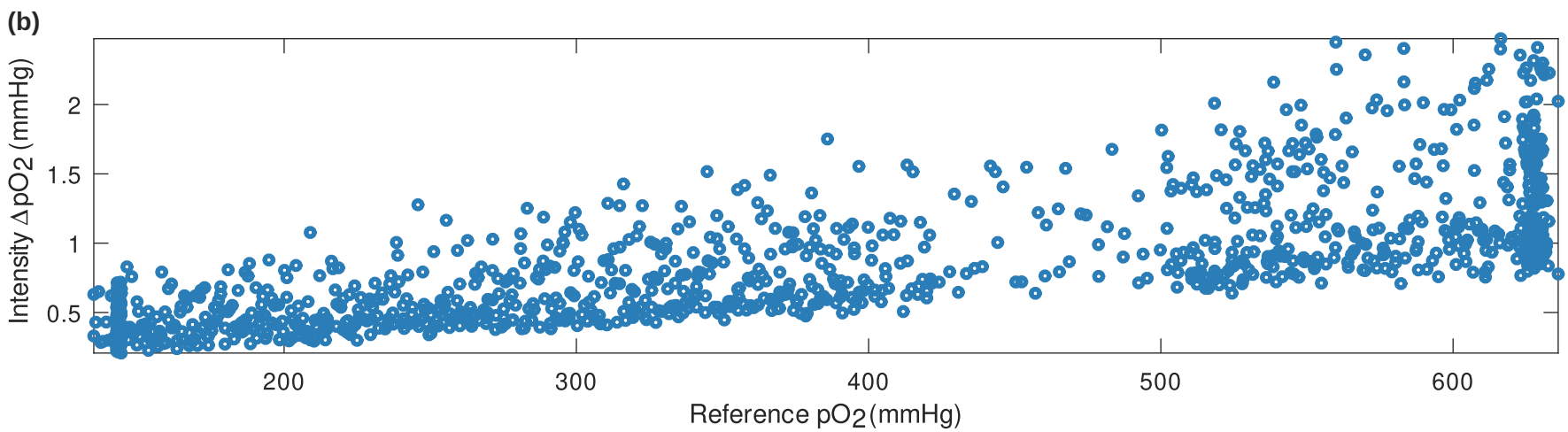

Supplement: SD-003-D3SD00240C-s002 [file SD-003-D3SD00240C-s002.zip › FigS4.pdf]

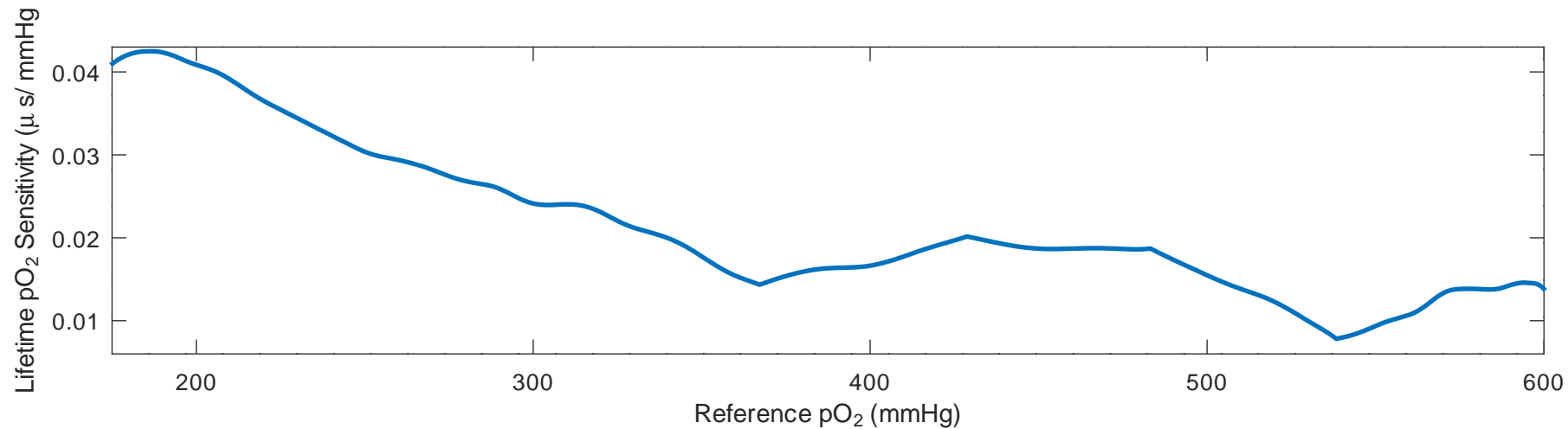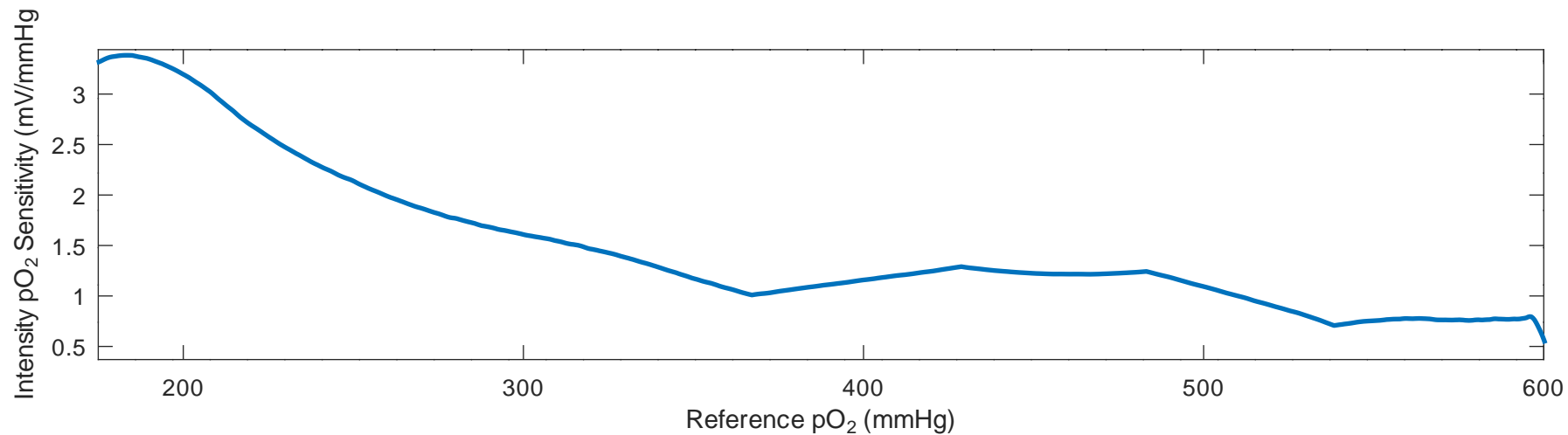

Supplement: SD-003-D3SD00240C-s002 [file SD-003-D3SD00240C-s002.zip › FigS5.pdf]
